# Salicylic acid stabilizes *Staphylococcus aureus* biofilm by impairing the *agr* quorum-sensing system

**DOI:** 10.1038/s41598-021-82308-y

**Published:** 2021-02-03

**Authors:** Cristian Dotto, Andrea Lombarte Serrat, Martín Ledesma, Carlos Vay, Monika Ehling-Schulz, Daniel O. Sordelli, Tom Grunert, Fernanda Buzzola

**Affiliations:** 1 Instituto de Investigaciones en Microbiología y Parasitología Médica (IMPaM), Consejo Nacional de Investigaciones Científicas y Técnicas (CONICET), Universidad de Buenos Aires, Buenos Aires, Argentina; 2 Departamento de Microbiología, Parasitología e Inmunología, Facultad de Medicina, Universidad de Buenos Aires, Buenos Aires, Argentina; 3 Instituto de Fisiopatología y Bioquímica Clínica (INFIBIOC), Facultad de Farmacia y Bioquímica, Hospital de Clínicas José de San Martín, Universidad de Buenos Aires, Buenos Aires, Argentina; 4 Functional Microbiology, Institute of Microbiology, Department of Pathobiology, University of Veterinary Medicine, Vienna, Austria; 5 Instituto de Investigaciones en Ingeniería Genética y Biología Molecular “Dr. Héctor N. Torres” (INGEBI), CONICET, Buenos Aires, Argentina

**Keywords:** Biofilms, Bacterial infection

## Abstract

Salicylic acid (SAL) has recently been shown to induce biofilm formation in *Staphylococcus aureus* and to affect the expression of virulence factors. This study was aimed to investigate the effect of SAL on the regulatory *agr* system and its impact on *S. aureus* biofilm formation. The *agr* quorum-sensing system, which is a central regulator in *S. aureus* pathogenicity, plays a pivotal role in the dispersal of *S. aureus* mature biofilms and contributes to the creation of new colonization sites. Here, we demonstrate that SAL impairs biofilm dispersal by interfering with *agr* expression. As revealed by our work, protease and surfactant molecule production is diminished, and bacterial cell autolysis is also negatively affected by SAL. Furthermore, as a consequence of SAL treatment, the *S. aureus* biofilm matrix revealed the lack of extracellular DNA. In silico docking and simulation of molecular dynamics provided evidence for a potential interaction of AgrA and SAL, resulting in reduced activity of the *agr* system. In conclusion, SAL stabilized the mature *S. aureus* biofilms, which may prevent bacterial cell dissemination. However, it may foster the establishment of infections locally and consequently increase bacterial persistence leading to therapeutic failure.

## Introduction

*Staphylococcus aureus* is the causative agent of a wide variety of infections in humans and other mammals, owing mainly to the variability of the many virulence factors that it possesses. In particular, this pathogen has the ability to adopt the biofilm lifestyle on tissues and medical device surfaces this conferring several advantages such as evasion of host immunity, which may lead to infection chronicity^1^. Biofilm formation during chronicity is one of the staphylococcal strategies that contributes to the failure of antibacterial therapy^2^. Staphylococcal biofilm-associated infections are characterized by tolerance to clinically relevant pharmacokinetic and pharmacodynamic dosing of antibiotics regardless of the in vitro susceptibility of planktonic cells^3^. Biofilm formation occurs in three main stages: first, adhesion of bacterial cells to the surface followed by maturation of the biofilm and dispersion of some bacteria from the mature biofilm to new possible infection sites in the host^4^. During maturation, bacteria produce adhesive molecules that constitute the biofilm matrix. This includes in particular the main exopolysaccharide of the *S. aureus* biofilm matrix, polysaccharide intercellular adhesin (PIA), also referred as poly-N-acetylglucosamine (PNAG)^5,6^. Alternatively, surface proteins, such as biofilm-associated protein (Bap), surface protein G (SasG), extracellular adherence protein (Eap), and fibronectin binding proteins (FnBPs), can contribute to intercellular adhesion^7^. In addition, extracellular DNA (eDNA) released by bacterial autolysis can be an important component of the biofilm matrix^8^. Biofilm dispersion promoted by dispersant factors, such as proteases, DNAses, and surfactant molecules as for instance phenol-soluble modulin (PSM) peptides released by *S. aureus* from the biofilm contributes to bacterial cell dissemination and the settlement of new biofilms at a distant site^9,10^. The progress through these stages involves the interplay of a complex regulatory network^11^. In particular, the *agr* system is an important regulator for initial attachment and biofilm disassembly by repressing the adhesins and stimulating the dispersal factors such as proteases and PSMs^12^. The coordinated expression of these factors is essential to ensure the progress along the three stages of biofilm formation and to develop *S. aureus* chronic infections.

The *agr* locus is composed of two transcriptional units, which are expressed from two divergent promoters. The P2 promoter controls *agrBDCA* transcription (RNAII transcript) whose products are involved in bacterial quorum sensing. AgrC and AgrA are the histidine kinase and the response regulator proteins, respectively, of a bacterial two components system. AgrD is the autoinducer peptide (AIP) precursor of the system, and AgrB is a transmembrane protease that processes AgrD to form the functional, extracellular AIP. Moreover RNAIII, the intracellular effector of the quorum-sensing system, is transcribed from the P3 promoter and encodes for δ-hemolysin (also called Hld), a cytolytic peptide belonging to the PSM family^13^. The AgrA transcriptional factor induces RNAII and RNAIII transcription from both, P2 and P3 promoters, and PSM peptides^14^, along with proteases and δ-hemolysin are important biofilm dispersal factors^15^.

Different environmental factors modulate biofilm formation by *S. aureus*^16^. During infection, certain pharmacological agents or their derivatives may become a signal for bacteria to promote regulatory changes, and consequently alter gene expression thus allowing *S. aureus* adaptation to persist in the host. Acetylsalicylic acid was designated by the World Health Organization as an essential agent because of its beneficial therapeutic role as analgesic but also as a preventive agent of heart attack and vascular brain accident. Indeed, recently the GLOBAL LEADERS trial has suggested that aspirin should be considered the preferred antiplatelet therapy for prevention of myocardial infarction and even ischaemic stroke^17^. Once ingested, aspirin is converted to salicylic acid (SAL) which is the metabolite responsible for the anti-inflammatory, antipyretic, and antithrombotic properties in humans^18^. Recently, we demonstrated that SAL strongly promotes *S. aureus* biofilm formation regardless of the methicillin susceptibility or clonal genomic characteristics^19^. In fact, the moderate iron-chelating ability of SAL induced biofilm formation in a PIA dependent manner and involved several metabolic, and regulation pathways in *S. aureus*^19^. Moreover, the production of a number of bacterial virulence factors is affected by SAL^20^. Interestingly, aspirin inhibits *Pseudomonas aeruginosa* quorum sensing by diminishing the production of certain virulence factors such as adhesins, biofilm and toxins^21^. The present study demonstrates that SAL impairs biofilm dispersion by interfering with *agr* expression. Consequently, the production of extracellular proteases and surfactants (PSMs and δ-hemolysin) was diminished. SAL can stimulate biofilm formation by *S. aureus*^19^ and, also, stabilize it by preventing bacterial dispersion from the biofilm to different sites in host.

## Results

### SAL impairs biofilm dispersion by affecting *agr*

**Figure 1:**
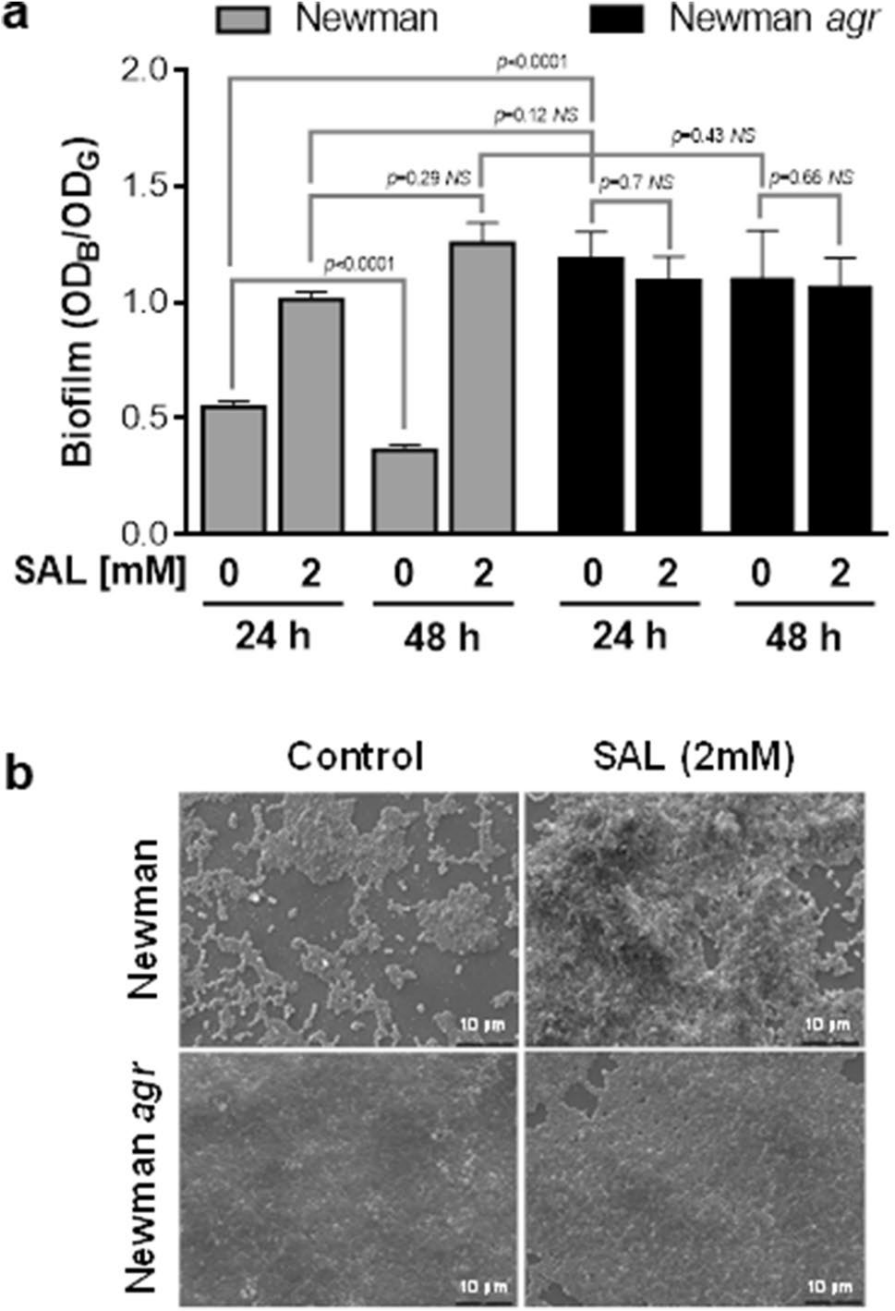
SAL prevents *S. aureus* biofilm dispersion. (**a**) Biofilm formation by the Newman strain or Newman *agr* mutant exposed to 2 mM of SAL measured after 24 and 48 h of static incubation at 37 °C. Each bar represents the mean ± SEM of 8 wells from 4 independent experiments. Biofilm formation values correspond to the OD_595_ of crystal violet (OD_B_) measured relative to the final culture density (OD_G_) after 24 or 48 h incubation. Comparison of Newman treated with SAL and Newman *agr* treated with SAL at both time points showed no significant differences. Statistical analysis was performed with the Mann–Whitney test. (**b**) Scanning electron microscopy (SEM) representative images of Newman and Newman *agr* biofilms grown statically for 24 h at 37 °C in the presence or absence of 2 mM of SAL. The black bars correspond to 10 μm. The magnification is 2000×.

Newman strain biofilms were quantified after 24 and 48 h exposure to SAL at a 2 mM concentration. In agreement with the results from our previous studies^19^, the biofilm formation was significantly enhanced by SAL at both time points. However, the amount of biofilm remained constant over time when SAL was present (Fig. 1a). In contrast, the biofilm biomass detectable after 48 h in the absence of SAL was significantly reduced (Fig. 1a). Although the SAL caused a slight delay in the growth of the Newman and Newman *agr* strains in short times, the growth was similar in all cases after 24 h (Supplementary Fig. S1). The number of viable *S. aureus* cells leaving biofilm at 48 h was slightly major than that observed at 24 h (Table 1). Indeed, the number of bacterial cells released from the biofilm seems to decrease in a SAL concentration-dependent manner. The impaired dispersal in the presence of SAL was observed in the drastic drop of planktonic biomass concomitant with the increment of biofilm biomass at 24 and 48 h (Supplementary Fig. S2). The SEM images of the biofilm formed by Newman showed abundant aggregation of bacterial cells in the presence of SAL (Fig. 1b).

**Table 1:** Bacteria dispersed from biofilm.

*S. aureus* treated with	Free^‡^ CFU/ml (× 10^6^) 24 h	Free^‡^ CFU/ml (× 10^6^) 48 h
0 mM SAL	8.63 (100%)	29.1 (100%)
0.36 mM SAL	2.42 (28%)	3.69 (12.7%)
2 mM SAL	0.17 (1.9%)	0.02 (0.07%)

^**‡**^Number of floating viable bacterial cells from biofilm grown during 24 or 48 h in the presence or absence of SAL normalized to the initial CFU/ml.

Since the Agr system promotes biofilm dispersion in *S. aureus*^15^, the ability to form biofilm in the presence of SAL by the Newman *agr-*defective mutant was studied. As expected, the Newman *agr* mutant was able to form more robust biofilms than the wild type (Fig. 1a). The presence of SAL did not produce appreciable changes in the architecture of the biofilm formed by the Newman *agr* strain as obtained by SEM (Fig. 1b). In addition, the biofilm biomass formed by the Newman *agr* mutant exposed to SAL revealed no significant changes compared to untreated conditions at both time points (Fig. 1a). Interestingly, the *agr* mutant produced a similar amount of biofilm than that observed with the wild type strain grown with SAL.

**Figure 2:**
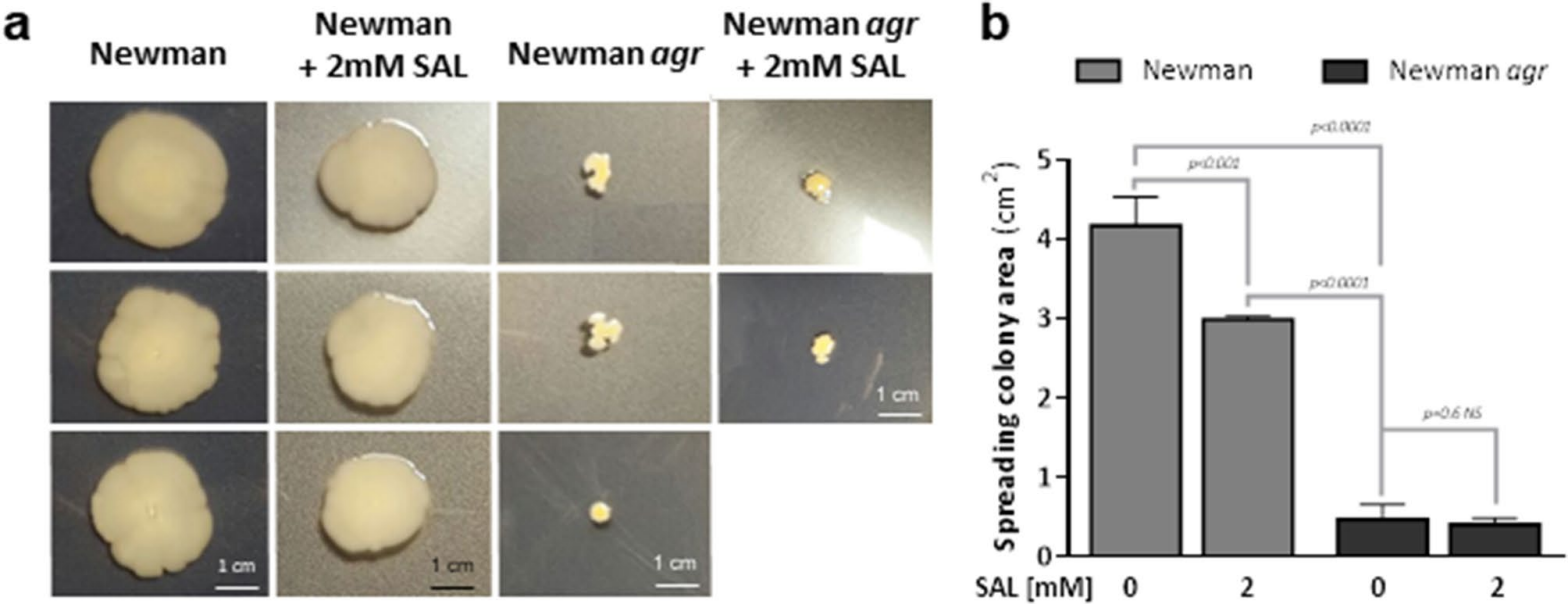
SAL affects *S. aureus* spreading colonies ability. (**a**) The spreading colonies from overnight cultures cultured with or without 2 mM of SAL spotted in the middle of LB soft agar plates supplemented or not with 2 mM of SAL. Pictures are representative of three independent experiments with three replicates each. After 24 h of incubation at 37 °C, the plates were photographed and (**b**) the spreading colony areas (cm^2^) were determined using ImageJ software^65^. Each bar represents the mean ± SD from 3 independent experiments. Statistical analyses were performed with one-way ANOVA and Holm’s test for multiple comparisons.

Next, the effect of SAL on colony spreading was studied since the Agr system is known for its positive regulation^22^. The exposure of *S. aureus* to 2 mM of SAL provoked a moderate reduction of the area of colony spreading by the Newman strain (Fig. 2a,b). A much more pronounced reduction of the colony spreading in presence or not of SAL was seen when the *agr* system was absent in comparison with those observed with Newman treated or not with SAL. In contrast, in biofilm, no differences were observed among Newman plus SAL and its *agr* mutant treated or not with SAL (Fig. 1a). This observation suggested that SAL may differentially affect biofilm and colony spreading. For colony spreading, the effect of SAL can only be partially explained by *agr*.

Collectively, these results suggest that SAL has a negative effect on *agr* function, thus reducing the colony spreading and impairing dispersion of mature biofilms.

### SAL reduces hemolysis and proteolysis activity of the biofilms

Proteases and δ-hemolysin contribute to the disassembly and dispersal of biofilms stimulated by *agr* via RNAIII. Thus, hemolytic and proteolytic abilities of the biofilm supernatants were analyzed. Supernatants of mature biofilms grown with SAL exhibited low α- and δ-hemolytic activities as revealed by the reduced diameters of the hemolytic halos on rabbit and sheep blood agar plates, respectively (Table 2, Supplementary Fig. S3). These results suggest that production and export of α- and δ-hemolysins to the extracellular medium from biofilms were decreased in the presence of SAL. Similarly, proteolysis halos of smaller diameter were observed when the same supernatants were plated on milk agar plates (Table 2). In both cases, the Newman *agr* mutant was used as a negative control of hemolysis and proteolysis. These results suggest that SAL has a negative impact on proteases production, thus resulting in a high content of surface proteins in the biofilm extracellular matrix. Besides, the low expression of δ-hemolysin by the effect of SAL would contribute to diminishing the disaggregation of the biofilm matrix.

**Table 2:** Hemolytic and proteolytic activity of supernatants of *S. aureus* biofilms.

Supernatants of mature biofilms	Diameter of hemolysis halo (mm)		Diameter of proteolysis (mm)
	α-hemolysin	δ-hemolysin	
Newman	3.00 ± 0.06	3.33 ± 0.17	7.7 ± 0.2
Newman + 2 mM SAL	2.50 ± 0.06 (*)	2.17 ± 0.17 (*)	4.5 ± 1.2 (*)
Newman *agr*	0	0	0

Asterisks represent the significance of the decrease in the hemolysis and proteolysis abilities mediated by SAL (*t-*test).

### SAL induces changes in the composition of biofilm proteins

**Figure 3:**
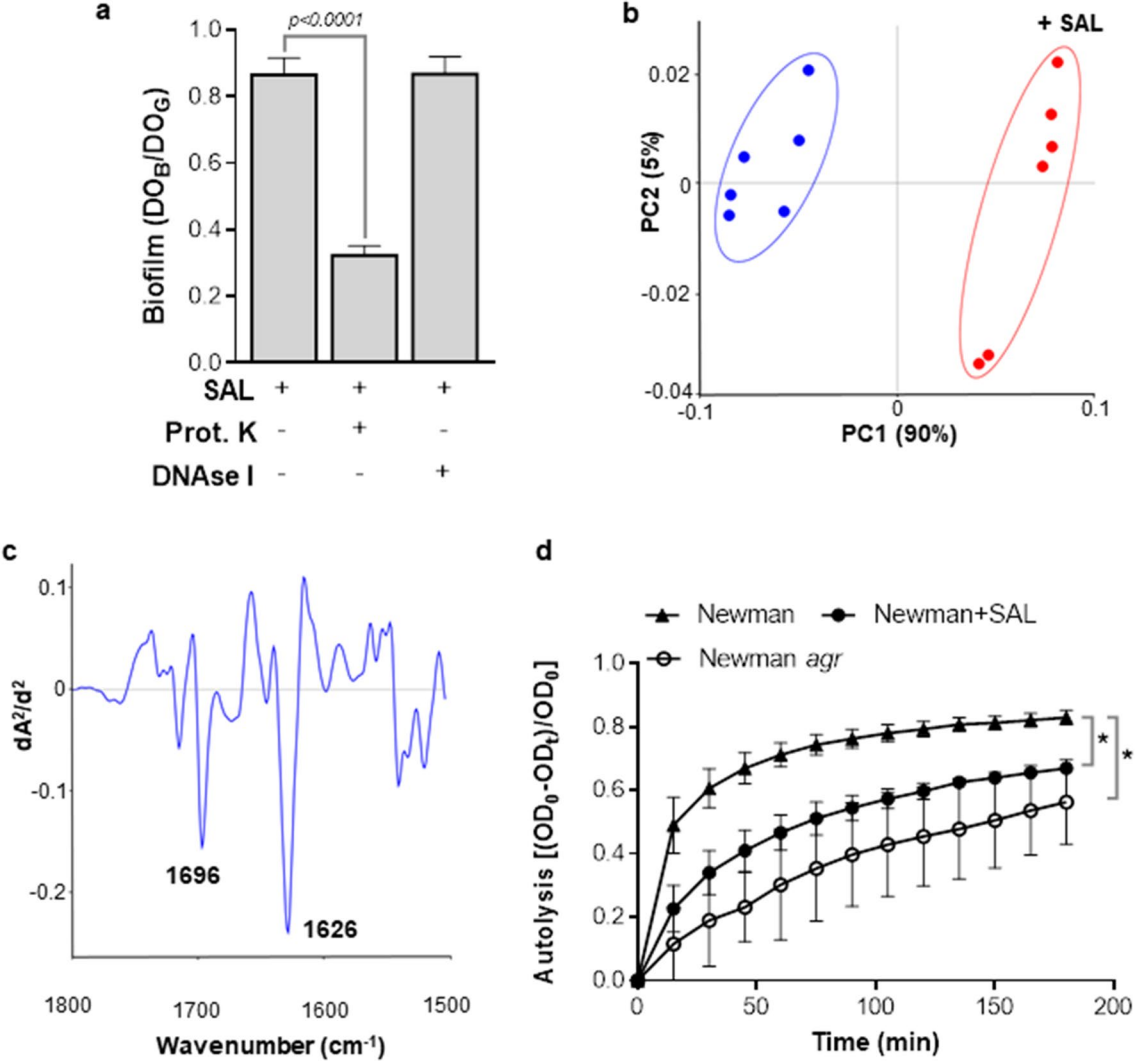
SAL alters the extracellular matrix components and autolysis. (**a**) Treatments with proteinase K (prot. K) or DNAse I of Newman biofilms formed for 24 h with 2 mM of SAL. Biofilm formation values correspond to the OD_595_ of crystal violet (OD_B_) measured relative to the final culture density (OD_G_) after 24 h incubation. Each bar represents the mean ± SEM of 6–8 wells from 3 to 4 independent experiments. Statistical analysis was performed with the Mann–Whitney test. (**b**) Principal component analysis (PCA) was carried out using the second derivative of vector normalized FTIR spectra in the range corresponding to amide bonds (1500–1800 cm^−1^) recorded from Newman biofilms. Biofilms were formed in TSBg with 2 mM of SAL during 24 h (red symbols) or without SAL (blue symbols). Different groups were indicated by ellipses in the figure. The relative contribution of each principal component is indicated in parenthesis. **(c)** Loading plot of SAL effect on *S. aureus* biofilm in the spectral region for protein constituents (1800–1500 cm^−1^). Spectral changes at 1626 cm^−1^ and 1696 cm^−1^ are related to alterations in the protein’s β-sheet conformation as well as anti-parallel pleated sheets and β-turn structures, respectively. **(d)** The Newman and Newman *agr* strain cultures were grown in TSBg with or without 2 mM of SAL to exponential phase. Bacterial lysis mediated by Triton X-100 was measured spectrophotometrically as a decrease in OD_600_ over time. Autolysis was defined as Eq. 1. The curves represent 2 independent experiments, with significant changes in comparisons: control *vs* SAL treatment and wild type *vs agr* mutant, denoted with asterisks (*t-*test).

Proteinase K treatment of Newman biofilm formed with SAL was carried out. This treatment caused 63% detachment indicating a high protein content in the extracellular matrix (Fig. 3a). Possible qualitative changes of *S. aureus* biofilm proteins due to SAL were studied by means of FTIR spectroscopic fingerprinting. Spectral data of biofilms formed in the presence of SAL were compared with those of untreated samples and analyzed in the spectral range corresponding to amide bonds (1800–1500 cm^−1^). The principal component analysis (PCA) revealed separate clusters at PC-1 (90%), indicating remarkable qualitative and quantitative differences in the protein composition between SAL-treated and untreated biofilm matrices (Fig. 3b). The observed clustering is mainly associated with spectral changes at 1626 cm^−1^ and 1696 cm^−1^ (Fig. 3c), which are described to reflect alterations in the protein’s β-sheet conformation as well as anti-parallel pleated sheets and β-turn structures, respectively^23^. Thus, due to the important contribution of proteins to the extracellular matrix, the profile of surface proteins in biofilms treated with SAL was investigated by Coomassie-stained SDS-PAGE (Supplementary Fig. S4). Most remarkably, a slight increased abundance of the adhesins Eap, Emp and Ssl7, as identified by MALDI-TOF–MS/MS, was observed in SAL treated biofilms (Supplementary Table 1). In regard to these results, it has been previously shown that the expression of Eap, Emp, and Ssl7 proteins are down regulated by *agr*^24,25^.

### SAL decreases autolysis and impairs eDNA release

Bacterial autolysis promotes eDNA release and its accumulation in the extracellular matrix of biofilms, hence the eDNA content was analyzed in *S. aureus* biofilms formed in the presence of SAL. Enzymatic action of DNAse did not alter the quantity of the biofilm biomass grown with SAL (Fig. 3a). However, despite low DNA from Newman biofilms, its concentration declined slightly in a SAL concentration-dependent manner (Newman: 10.2; Newman + 0.36 mM SAL: 9.0; Newman + 2 mM SAL: 8.1; and Newman + 5 mM SAL: 6.9 ng/ml of eDNA). Next, autolysis by Triton X-100 in planktonic cultures of the Newman strain exposed to SAL was investigated since it was shown that the activation of *agr* locus induces bacterial autolysis^26^. Indeed, the Newman strain exposed to SAL showed a significant lower autolysis rate compared with untreated conditions (Fig. 3d). The Newman *agr* mutant was used as a negative control. Taken together, these results show that SAL promotes a decrease in autolysis and diminishes eDNA concentration in biofilms.

### In silico interaction of SAL and AgrA

**Figure 4:**
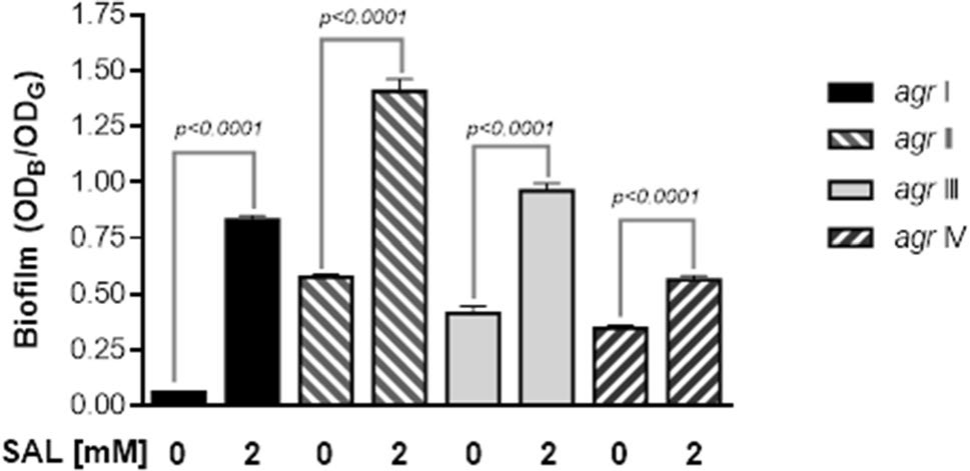
SAL promotes biofilm formation among *S. aureus* strains of *agr* types I to IV. Biomass quantification of the biofilms generated by the *S. aureus* BRA (*agr* I), CBS (*agr* II), MW2 (*agr* III) and RN8540 (*agr* IV) strains formed for 24 h at 37 °C in the presence or absence of 2 mM of SAL. Each bar represents the mean ± SEM from 4 independent experiments with 8 wells for each condition. Statistical analysis was performed with the Mann–Whitney test.

Based upon the region of hyper-variability within *agrBCD*, *S. aureus* strains are classified as *agr* type I to IV, which led us to hypothesize that the impact of SAL on biofilm could be *agr* type-dependent. As shown in Fig. 4, the effect of SAL on increment of biofilm was observed in all *agr* types. Therefore, we speculate that the effect of SAL on AgrA is of general nature and might be explained by the effect that it targets a conserved region within this regulator.

**Figure 5:**
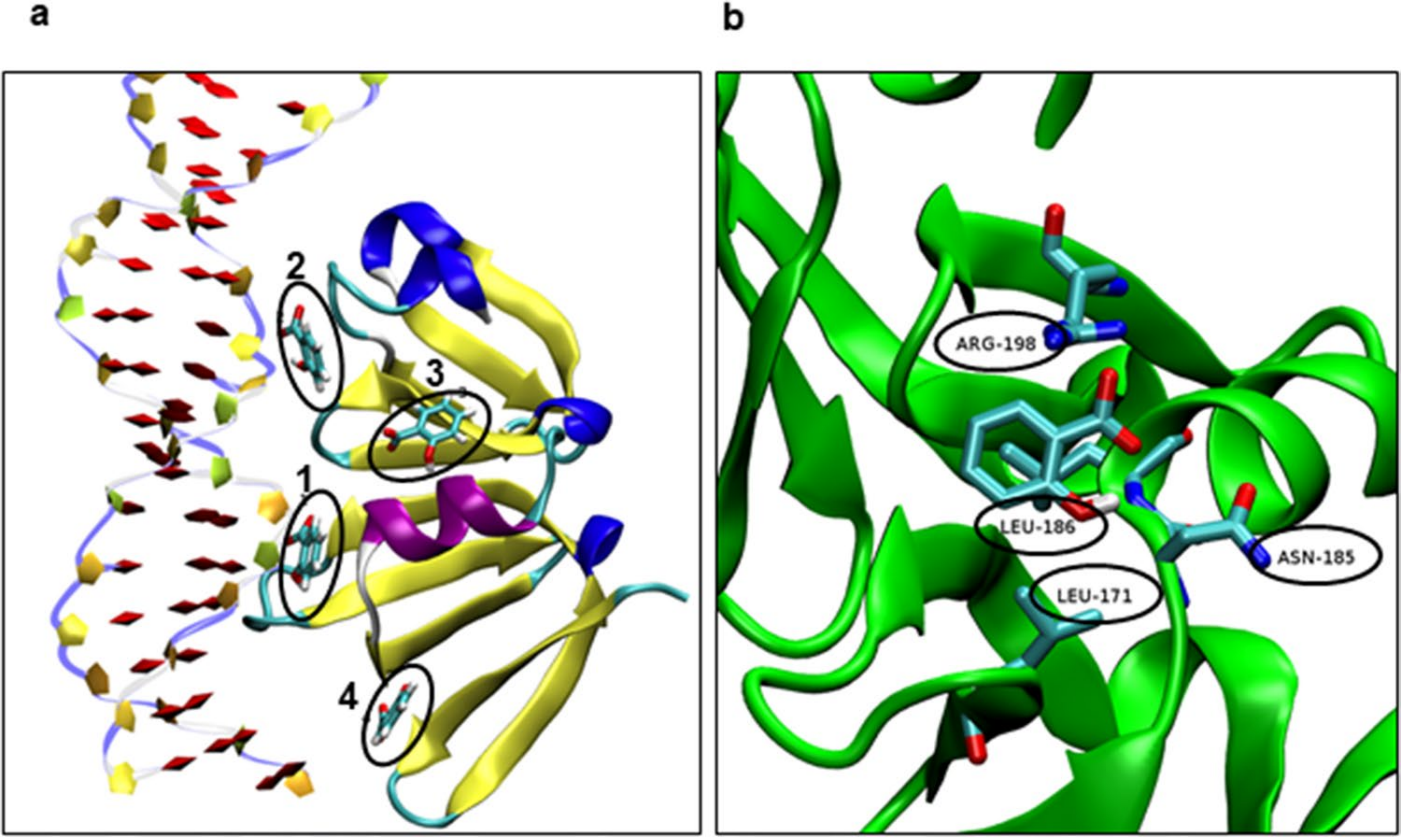
In silico determination of potential interaction sites on AgrA with SAL. (**a**) The figure depicts 4 interaction sites between SAL and AgrA overlapping a DNA fragment, determined by the AutoDock4 v4.2.6 and AutoDock Vina v1.1.2 softwares^28,29^. The AgrA regulator is shown with a “New Cartoon” representation with α-helixes, β-sheets and loops are represented in magenta, yellow and blue colors, respectively. The DNA fragment is shown with a “PapeChain” representation with phosphates groups, deoxyribose residues and nitrogen bases shown in blue, yellow and red colors, respectively. SAL is shown in each site (1–4) with an ellipse, with a “Licorice” representation with C, O and H atoms shown in cyan, red and white colors, respectively. (**b**) The figure depicts the predicted complex between AgrA and SAL in site 1, with the main amino acid residues involved in the interaction denoted by ellipses (Leu-186, Leu-171, Arg-198, and Asn-185).

SAL is a small molecule able to penetrate into cells and interact with transcriptional factors and DNA^27^. Thus, the potential interaction between SAL and the AgrA protein (DNA interaction domain, PBD ID: 3BS1) was evaluated in silico performing a docking assay. Four potential sites of interaction between AgrA and SAL with different interaction energies were identified using AutoDock4 v4.2.6 (http://autodock.scripps.edu) and AutoDock Vina v1.1.2 (http://vina.scripps.edu) softwares^28,29^ (Fig. 5a). Sites 1 and 2 are placed in regions of AgrA that are involved in the interaction with DNA. Small pockets can be formed in those sites where SAL can be placed. Moreover, the non-polar SAL portion is positioned to Leu residues and the carboxylate to positively charged amino acids residues. In particular, in site 1, SAL is oriented towards the Arg198 residue which is next to a Cys residue involved in the redox mechanism that lead AgrA-DNA interaction^30^ (Fig. 5b). SAL in sites 3 and 4 would not interfere with the linkage between the transcriptional factor and DNA since both sites are at some distance from the DNA interaction site. Particularly, SAL in site 3 is localized on the α-helix of the regulator, exposed to the solvent and far away from the DNA interaction site.

To evaluate the stability of SAL in the four sites identified by molecular docking, a simulation of molecular dynamics was performed. A simulation with AgrA in absence of SAL was performed as a control, and fluctuations due to the protein backbone were detected over time (Supplementary Fig. S5a). This result indicated that the regulator reaches the stability at approximately 50 ns of simulation after two small fluctuations which are expected if considering the protein structure was obtained by X-ray diffraction. There were not significant differences in the protein conformation when SAL was present, showing only values that correspond to the protein. Moreover, SAL remained without significant variations over simulation only when it was positioned in site 4, showing a 180° inversion with regard to the molecule minor axis at the 65 ns of simulation (Supplementary Fig. S5b). This fluctuation did not affect the AgrA-SAL interaction since it involves mainly the SAL aromatic group and the Tyr183 residue of AgrA. This interaction was determined from an overlapping of sequential image capture during 15 ns, where SAL adopted a conformation with the hydroxyl group up for to the half of that time (Supplementary Fig. S6).

### SAL downregulates *agr* and *psm* expression

**Figure 6:**
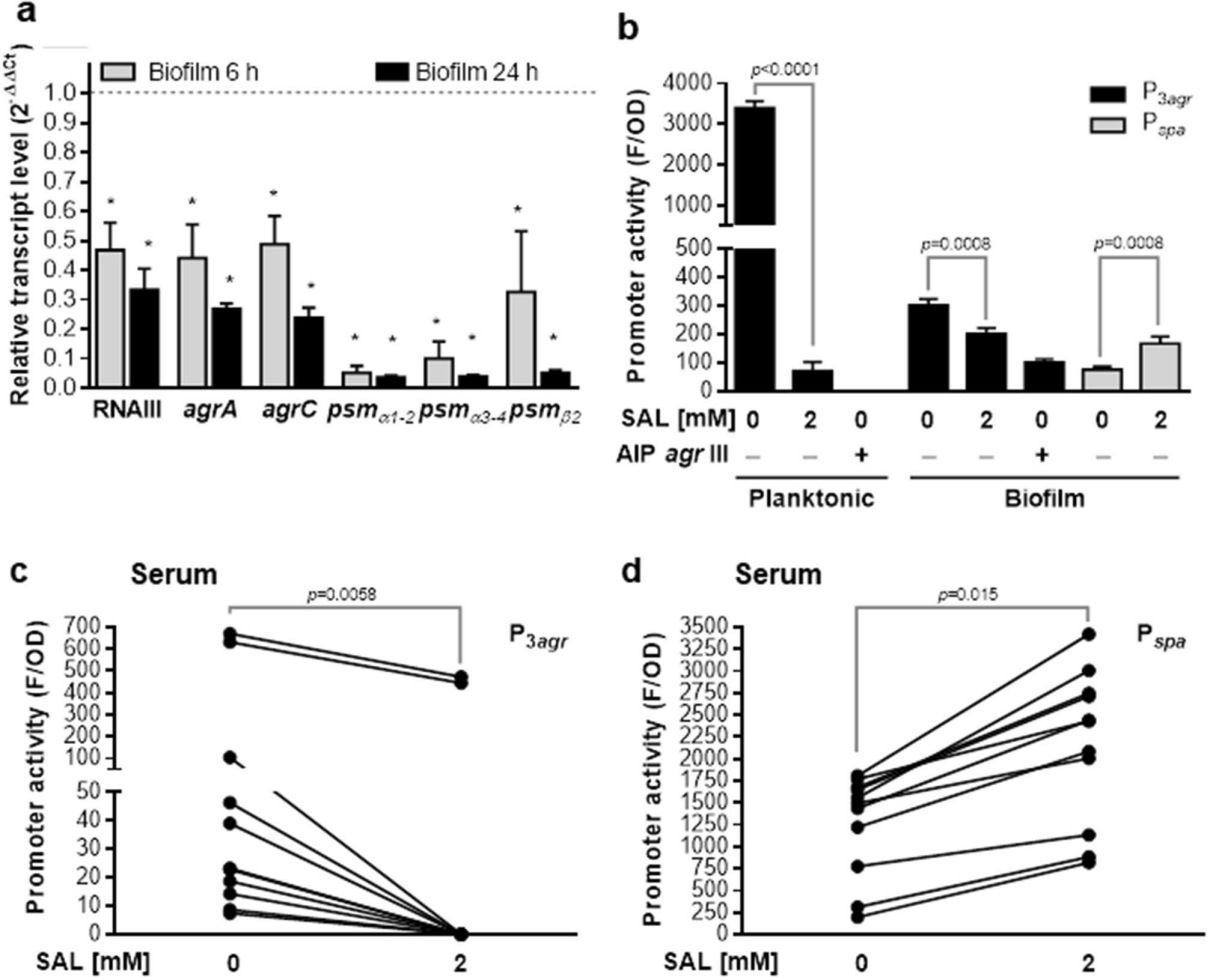
SAL modifies *agr, psm* and, *spa* transcription. (**a**) RNAIII, *agrA*, *agrC*, *psmα*_*1-2*_, *psmα*_*3-4*_ and *psmβ*_*2*_ transcripts were quantified in immature (6 h) and mature (24 h) Newman biofilms formed in TSBg with 2 mM SAL. Genic expression changes were informed as fold changes (2^−∆∆Ct^). Data were related to the expression of the housekeeping gene *gyrB*. Values of 2^−∆∆Ct^ < 1 represent a decreased expression with SAL treatment. Each bar represents the mean ± SEM from duplicates of 3 independent experiments. The asterisk symbol means a significant change (*t-*test). (**b**) Planktonic and biofilms cultures of Newman_P3*agr*_ and Newman_P*spa*_ grown for 24 h in TSBg with 2 mM of SAL or supernatants of *S. aureus agr* type III cultures, enriched in AIP type III. Promoter P_3*agr*_ y P_*spa*_ activities were determined by *gfp* fusion and quantification of fluorescence (F) corresponding to GFP. F values were related to the final growing optical density (OD). Each bar represents the mean ± SEM of 6–8 wells from 3 to 4 independent experiments. Statistical comparisons are represented by lines with the *p* values denoted above (*t-*test). (**c**) Eight-hour planktonic cultures of Newman_P3*agr*_ and (**d**) Newman_P*spa*_ strains grown in human serum from 11 donors with 2 mM of SAL. The lines link each donor represented with a dot, in both conditions and the *p* values denoted above (*t-* test).

Because SAL could interfere with the interaction of AgrA and the genes under its control, the transcriptional profile of genes stimulated by AgrA was analyzed by RT-qPCR. Initially, the level of *psm*_*α*_ transcripts was quantified in biofilms formed by the Newman strain and its isogenic *agr-*defective mutant. The results showed downregulation of *psm*_*α*_ in the *agr* mutant (2^−∆∆Ct^_*psmα*1-2_: 0.0014 ± 0.0006; 2^−∆∆Ct^_*psmα*3-4_: 0.1260 ± 0.0720; *p* < 0.0001) under the experimental conditions used. To get an insight about the transcriptional state of the *agr* locus in biofilms exposed to SAL, the *agrA*, *agrC* and *psm* transcripts were quantified by RT-qPCR. The presence of SAL promoted a significant decrease in the levels of transcripts *psmα*_1-2_, *psmα*_3-4_ and *psmβ*_2_ both in immature (6 h) and mature (24 h) *S. aureus* biofilms (Fig. 6a). Indeed, RNAIII, *agrA* and *agrC* transcripts were also downregulated in biofilms formed with SAL (Fig. 6a). Also, the activity of the P3_*agr*_ promoter was significantly decreased by SAL in both planktonic cultures and mature biofilms and SAL enhanced the P_*spa*_ promoter activity in biofilm lifestyle, which is downregulated by *agr*^31^ (Fig. 6b). Furthermore, the decrease in promoter activity induced by SAL resembled that achieved by the addition of AIP type III, an *agr* I inhibitor used as a negative control. It should be noted that the Newman strain possesses an *agr* type I^3^. These results, together with the in silico prediction, suggest that the steric hindrance generated by SAL (Supplementary Fig. S6) may prevent an appropriate interaction of AgrA and its target promoters, such as P3 from the *agr* locus. Owing to the downregulation exerted by SAL at the transcriptional level of *agr* and *psm* genes, a proteomic approach was carried out. The results of MALDI-TOF–MS showed that SAL reduces the abundance of AgrD, PSMα3, PSMβ1 and δ-hemolysin (encoded by RNAIII) and increase SpA translation in Newman colonies (Table 3).

**Table 3:** *S. aureus* proteins affected by SAL.

Protein identified	Accesion number	Role in biofilm development	SAL effect	Stimulated by	*t*-score
PSMβ1	H9BRQ3	Dispersion	Down	AgrA	− 2.449
δ-hemolysin	P0C1V1	Dispersion	Down	AgrA	− 2.449
PSMα3	P0C812	Dispersion	Down	AgrA	− 2.449
AgrD	Q9L561	Dispersion	Down	AgrA	− 2.449
SpA	A0A346CG97	Adhesion and maduration	Up	RNAIII	2.449

Proteins identified by MALDI-TOF–MS. *t-*score values < − 2.015 or > 2.015 are significant.

To determine whether the presence of SAL in human serum was able to downregulate *agr*, the *S. aureus* the P3_*agr*_ promoter activity was investigated. Planktonic cultures of the Newman P3_*agr*_ strain showed a significant reduction of P3_*agr*_ activity when SAL was present in the human serum (Fig. 6c). Moreover, SAL increased P_*spa*_ promoter activity (Fig. 6d). In summary, the results show that SAL decreased *agrA* transcription both in mature and immature biofilms of *S. aureus*. This downregulation of the *agr* locus activator gene may promote the decrease of the levels of *agrC*, RNAIII and also *psm*_*α*1-2_, *psm*_*α*3-4_ and *psm*_*β*2_ transcripts, as well as, the P3_*agr*_ promoter activity in biofilms and planktonic cultures cultured in the presence of human serum.

## Discussion

A high percentage of the world population consumes aspirin due to its antithrombotic, anti-inflammatory, and analgesic properties^32^. SAL is the main metabolite obtained from aspirin deacetylation in vivo, reaching serum concentrations from 0.36 mM up to 2 mM^33^. Indeed, SAL decreased the antibacterial effect of platelets against *S. aureus*^34^ and several effects of SAL on bacterial virulence have been described^20,35–38^. We recently showed that SAL diminished intracellular Fe^2+^ levels of *S. aureus* cells forming biofilms by increasing their biomass and promoting PIA production^19^. Indeed, the expression of PIA is not under the control of Agr system^39^. Here, we demonstrate that SAL reduces *agr* transcription and thus, negatively affects the expression of genes controlled by AgrA and by RNAIII, which leads to a decrease of factors involved in biofilm disassembly. The attenuation of the *agr* quorum sensing due to the presence of SAL maintains the integrity of biofilm.

The Newman *agr* mutant formed a biofilm with more biomass than the wild type, which corroborates findings from previous studies^15,39^. This increase is attributed to the ineffective detachment of cells from mature biofilm^40,41^. As revealed by our work, SAL effects on biofilm formed by the Newman strain were mimicking those of its *agr* mutant. Also, the high biomass of SAL treated biofilm was maintained over time, evidencing that SAL impaired the dispersion of mature biofilm. The main transcripts of the Agr system (*agrA*, *agrC,* and RNAIII) and the genes stimulated by AgrA (*psm*) were downregulated in the presence of SAL. This effect was supported at the translational level by the decreased production of δ-hemolysin (with a consequent reduction of the hemolytic activity), PSMα3 and PSMβ1 which are translated from polycistronic RNAs along with the other PSMα and PSMβ, respectively. Additionally, the P_*spa*_ promoter expression was stimulated by SAL as well as the abundance of SpA, which could be attributed to RNAIII downregulation. These results foster the hypothesis that SAL negatively impacts *agr* expression.

Dastgheyb et al.^42^ demonstrated that low activity of *agr* and, consequently, the decrease in PSMs production caused a strong aggregation of *S. aureus* cells in synovial fluid. Indeed, the colony spreading ability of *S. aureus* was stimulated by a high amount of cell surface PSMα1-4^43^. In contrast, δ-hemolysin diminished colony spreading by inhibiting PSMα1-4 binding to the *S*. *aureus* cell surface^43^. In the present work, the moderate decrease of colony spreading activity by SAL action could be explained by the decrease in PSMα3 and δ-hemolysin production. It should be speculated that SAL might alter the balance between the extent of cell surface PSMα1-4 (stimulators) and δ-hemolysin (inhibitor) required for the colony spreading ability. The effect of SAL on biofilm was more evident because not only PSMs and δ-hemolysin, but also proteases were decreased by the SAL action on the Agr system. All these molecules participate in the dispersion process, unlike the colony spreading activity, in which only PSMα1-4 and δ-hemolysin are required^43^. On the other hand, PSM peptides are necessary for separating PIA molecules from the bacterial surface^42^. We previously reported that SAL increased the PIA production in *S. aureus* biofilms^19^. Here, the diminution of PSMα3 and PSMβ1 peptides by the effect of SAL add new evidence that explains the high aggregation of biofilm by inefficient dissociation of PIA molecules of the extracellular biofilm matrix.

The Agr system has a main role in the dispersal of biofilms by *S. aureus* because *agr* stimulates the expression of extracellular proteases^15,40^. FTIR spectroscopy analyses of *S. aureus* biofilms revealed strong differences in the qualitative and/or quantitative composition of biofilm matrix proteins between SAL treated and untreated conditions. In particular, the spectral changes at 1626 cm^−1^ suggests alterations in the β-sheet conformation of biofilm extracellular matrix proteins associated with misfolding and/or aggregation^44,45^. It is noteworthy that the low proteolytic activity caused an accumulation of proteins in the extracellular matrix of biofilms treated with SAL, as it was evidenced after proteinase K treatment of biofilms. Eap, Emp and, SsI7 were some of the proteins accumulated, which are expressed when decreasing *agr* activity^24,25^. Therefore, the integrity of extracellular matrix proteinaceous components of biofilm induced by SAL was maintained. Staphylococcal superantigens like proteins (Ssl) inhibit the recruitment of neutrophils and complement activation, thus contributing to immune system evasion^46^. Exposure of *S. aureus* to SAL may reinforce the intrinsic resistance to host defenses of biofilms by increasing Ssl7 expression (Supplementary Fig. S1 and Supplementary Table S1). Indeed, eDNA is a constituent of the extracellular matrices of *S. aureus* biofilms^8^. eDNA release through bacterial autolysis is stimulated by *agr*^26^. In this regard, our results agree with those of Fujimoto et al.^26^ who reported that SAL decreased autolysis of *S. aureus* cultures. Moreover, SAL treatment resulted in a slight diminution of eDNA present in Newman biofilm, even though DNAse treatment did not have a significant effect. It is suggested that *agr* downregulation mediated by SAL delays both autolysis and, consequently, eDNA release.

The antioxidant ability of SAL was explained by its tendency to form complexes with iron ions^19^, which can interfere with the Fenton reaction^47^. Indeed, the regulatory mechanism of AgrA is controlled by the bacterial redox status since this protein has two conserved Cys residues involved in its binding to DNA^28^. Moreover, George et al.^48^ demonstrated that PSMs induce the production of ROS. An increase of intracellular ROS may contribute to the disruption of the extracellular matrix in the *S. aureus* biofilm^49^. SAL downregulated *psm* transcription as well as PSMα3 and PSMβ1 production in *S. aureus* and decreased endogenous ROS levels as it was ascertained by the NBT assay (Supplementary Fig. S7). Thus, it is tempting to speculate that the AgrA-DNA binding might be affected by SAL by modulating the redox status related to a low PMS production and/or a decrease in free iron ions concentration^19^ available for the Fenton reaction. Furthermore, SAL and some of its derivatives can interact with bacterial transcription factors and with sequences of DNA with a high AT percentage^27,50,51^. Particularly, the consensus sequence recognized by AgrA is a pair of direct repeats [TA][AC][CA]GTTN[AG][TG] separated by a 12–13 bp spacer region that are found in the P2–P3 intergenic region of RNAIII and the *agr* operon^52^. Indeed, DNAse I protection assays has been shown that AgrA binds to AT-rich sequences (75%) in this region^52^.

The in silico analysis suggested that SAL can bind four sites in AgrA. Complementary studies of molecular dynamics showed that SAL would frequently occupy the site number 4 (Fig. 5, Supplementary Fig. S5). Although this site would not be involved in AgrA-DNA binding, SAL could exert a steric hindrance effect when it moves between the four different sites, thus hampering the interaction between AgrA and its consensus sequences in target genes. In this way, the expression of the AgrA-regulated genes can be affected. Consistent with this, the P3_*agr*_ promoter activity decreased in the presence of SAL to similar levels as those obtained with AIPIII, which is a natural inhibitor of the Agr type I system that avoids AgrA activation and consequently its binding to DNA. Further research is needed to prove whether SAL interferes with the AgrA-DNA interaction either by creating a redox imbalance or by posing a steric impediment.

Other authors^53,54^ reported that apolipoprotein B present in human serum is an *agr* expression inhibitor due to its ability to capture the AIP signal^55^. Our results showed that the P3_*agr*_ promoter activity was lower in serum than in TSBg and that SAL affected this promoter by exerting a negative effect. Moreover, AgrD (precursor of AIP) expression was downregulated by SAL in Newman colonies reinforcing the intrinsic effect of SAL regardless of serum inhibition, thus contributing to downregulation of *agr* quorum sensing. It can be hypothesized that aspirin would act synergistically with plasma components and create conditions in vivo that may promote *agr* downregulation. Therefore, a few *S. aureus* cells with an attenuated quorum sensing system that escape from the biofilm (Table 1 and Supplementary Fig. S2) may have the advantage of adhering to and forming new stabilized biofilm elsewhere. Further, the *agr* dysfunctionality caused by SAL would favor infection chronicity^56^. Since it is common in clinical practice to co-administrate antibiotics and aspirin to infected patients^57^, the maintenance of a stabilized biofilm by SAL would help *S. aureus* to resist the action of antibiotics despite its antibiotic susceptibility in vitro. In addition, SAL may also increase the frequency of mutations leading to antibiotic resistance^57^. In fact, the active metabolite of aspirin may certainly contribute to therapeutic failure against *S. aureus* in long-term infections. Owing to the growing emergence of pathogens resistant to antibiotics, novel strategies to avoid the above mentioned problem are urgently needed. Antivirulence agents like those that impact on quorum sensing are currently being investigated^58^. More research in this field is required, whereas the effect on *S. aureus* pathogenesis of SAL deserves future attention.

**Figure 7:**
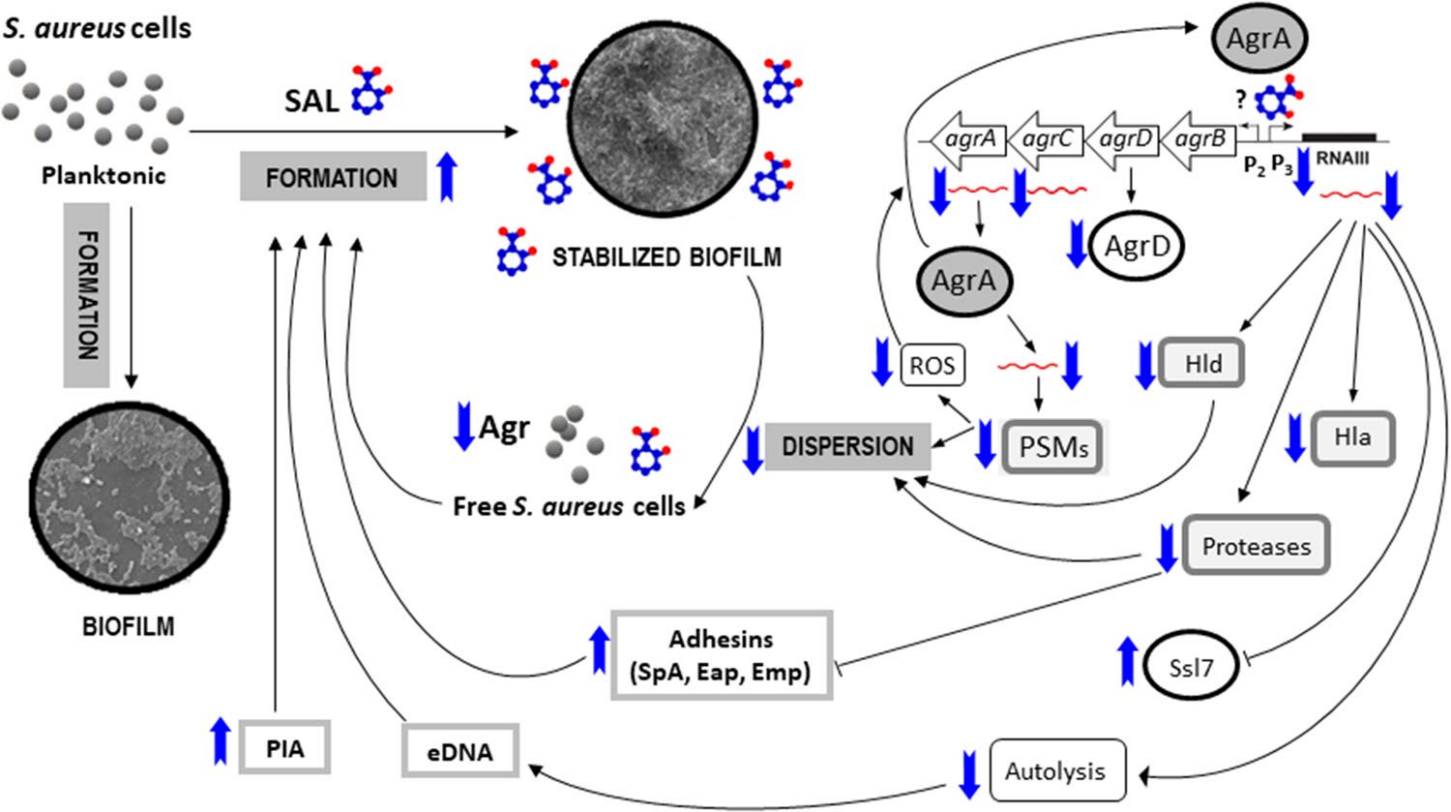
Schematic representation of SAL effects on stabilization of biofilm formed by *S. aureus*. Summary of changes determined in mRNA or protein expression, biochemical and phenotypic levels. Black lines with arrowheads endings denote stimulation and flat endings inhibition. Up and down blue arrows represent an increased or diminished change induced by SAL, respectively.

In conclusion, SAL impacts on the *agr* quorum sensing system (Fig. 7), limiting the bacterial cell escape from biofilm while maintaining high biomass in the mature biofilm, which can promote tolerance to antibiotics and make the infection refractory to the recommended antibiotic therapy. Our findings not only highlight that current treatment regimens must be reconsidered but also the opening of new roads for development of novel strategies to better cope with *S. aureus* infections.

## Materials and methods

### Bacteria and growth conditions

*Staphylococcus aureus* strains and plasmids used in this study are detailed in Supplementary Table 2. Bacterial strains were stored at − 80 °C in Trypticase Soy Broth (TSB) (Britania) supplemented with glycerol at a final concentration of 20%. Bacterial cultures were incubated at 37 °C in TSB added with 0.25% glucose (TSBg), Luria broth (LB) or human serum obtained from the blood bank of Universidad de Buenos Aires, Hospital de Clínicas “Gral. José de San Martín”, Buenos Aires, Argentina, under an existing Institutional Review Board protocol. Planktonic cultures were grown at 200 rpm for 18 h at 37 °C and the optical density (OD) at 600 nm was measured at the required times. SAL (Sigma-Aldrich) was added when necessary at the final concentration specified. Strains with plasmids or mutant strains were grown in culture media supplemented with the corresponding selection antibiotic.

### Biofilm formation

Two hundred µl/well of a 1/100 dilution of *S. aureus* cultures grown in TSBg for 18 h a 37 °C were loaded in polystyrene microplates (Greiner Bio-One). When widespread biofilms (for RNA and protein extraction) were needed, 75 cm^2^ polystyrene cell culture bottles (Nunc) were inoculated with 30 ml of a 1/100 dilution of an overnight culture. SAL was added at the concentrations indicated when necessary. After 6, 24 or 48 h of static incubation at 37 °C the biofilm biomass was quantitatively determined and RNA or proteins were extracted. Before biomass quantification, absorbance of each well, which represents bacterial growth, was determined spectrophotometrically by measuring the OD at 595 nm (OD_G_) with a microplate reader. Afterwards, each well was rinsed twice with PBS, the biofilms were fixed with methanol 100% for 15 min and stained with crystal violet 0.5% during 20 min. After eliminating the dye excess, the biofilms were suspended with 30% acetic acid. The biofilm biomass was quantified spectrophotometrically by measuring the OD at 595 nm (OD_B_). Each OD_B_ value was made relative to its corresponding OD_G_. *Staphylococcus aureus* biofilms for scanning electronic microscopy (SEM) were formed on a coverslip placed in wells of a 24 wells microplate (Greiner Bio-One). After static incubation at 37 °C for 24 h, biofilms were washed with PBS, fixed with formaldehyde 2.5% at 4 °C for 2 h and, dehydrated with 5 min incubations with ethanol in rising concentrations. Finally, the coverslip was positioned on aluminum support and covered with a metallic film of gold–palladium for a subsequent examination with a scanning electronic microscope Philips XL30 TMP.

### Biofilm dispersion

Biofilms of 24 h and 48 h formed in the presence of SAL and supernatants were transferred to sterile tubes. Biofilms were scraped and suspended in PBS. Dilutions of initial bacterial inocula and supernatants were plated on TSA, incubated at 37 °C for 18 h, and CFU/ml were determined.

### Enzymatic treatment of biofilms

Twenty-four hour biofilms were formed with or without SAL addition, and after washing with PBS, they were incubated for 2 h at 37 °C with 100 µg/ml proteinase K (Genbiotech) or 140 U/ml DNAse I (Promega) in Tris buffer 20 mM (pH 7.5). After treatment, biofilms were fixed, stained, and quantified spectrophotometrically.

### Biofilm analysis by Fourier transform infrared (FTIR) spectroscopy

Biofilms grown for 24 h were harvested by centrifugation at 10,000×*g* for 10 min and suspended in 100 µL PBS. 30 µl of the biofilm suspension was transferred to a ZnSe optical plate and dried for 40 min at 40 °C. FTIR spectra were acquired using a FTIR Tensor 27 spectrometer coupled to the microplate adapter HTS-XT (Bruker Optics GmbH). The infrared spectra were recorded in transmission mode in the spectral range between 4000 and 500 cm^−1^ using the spectrometer parameter as described elsewhere^59^. For principal component analysis (PCA), vector-normalized, second derivative spectra were computed and scores/loading plots were performed at the spectral region for protein constituents (1800–1500 cm^−1^) by using the Unscrambler X software (Camo analytics, v11.0; https://www.camo.com/spectroscopy/^44^.

### Autolysis induced by Triton X-100

Bacterial cells from exponential phase cultures of *S. aureus* grown with SAL were recovered by centrifugation at 10,000×*g* and 4 °C for 10 min and washed with sterile distilled H_2_O. Later, the washed pellet was suspended in lysis buffer A (0.05 M Tris–HCl pH 7.4 and 0.05% Triton X-100) and incubated at 30 °C and 200 rpm. Bacterial lysis was determined spectrophotometrically by measuring the OD_600_ every 15 min for 3–5 h. The autolytic ability was recorded as the relationship detailed in Eq. (1), where OD_0_ and OD_t_ are the initial OD and the OD measured at any given time, respectively.

1 $$ {\text{Autolysis }} = \, \left( {{\text{OD}}_{0} - {\text{ OD}}_{{\text{t}}} } \right)/{\text{OD}}_{0} $$

### Quantification of eDNA

eDNA from biofilms were quantified as described in Kaplan et al.^60^ with some modifications. Biofilms were grown in a 6-well polystyrene microtiter plate in 1 ml of TSBg supplemented with different SAL concentrations. After 24 h of growth, planktonic cells were carefully removed, and 1 ml of TE buffer (10 mMTris, 1 mM EDTA [pH 8]) was added to each well. The biofilm biomass was totally scrapped, transferred to a 1.5-ml microcentrifuge tube, and centrifuged at 13,000 rpm for 25 s to remove the supernatant. Biofilms pelleted were resuspended in 200 µl of TE buffer, and the tubes were again centrifuged. After eDNA precipitation with (NH_4_)_2_SO_4_ 2 M and cold EtOH 80% treatment overnight, the eDNA was recovered by centrifugation, dried at room temperature, and resuspended in 30 µl of distilled water. The concentration of eDNA was quantified spectrophotometrically at 260 nm.

### RNA extraction and reverse transcription quantitative real time PCR (RT-qPCR)

*Staphylococcus aureus* biofilms were formed on 75 cm^2^ surface of cell culture flasks (Nunc) over 6 or 24 h. After removing culture media, biofilms were washed with PBS, collected with a sterile scraper and transferred to an Eppendorf tube containing 1 ml of PBS. Later, bacterial cells were recovered by spinning the suspension at 10,000×*g* for 10 min. To obtain total RNA, bacterial cells were chemically lysed by incubation with 100 µl of lysis buffer B [Tris–EDTA 10:1 (10 mM Tris–HCl pH 8; 1 mM EDTA), 40 µl of 1 mg/ml lysostaphin, and 20 µl of 50 mg/ml lysozyme] at 37 °C for 20 min. RNA was extracted from lysates with the Trizol reagent (Invitrogen Life Technologies) according to the supplier directions. DNA was eliminated by RNAse free-DNAse RQ1 (Promega) treatment.

cDNA synthesis was performed with ImProm-II reverse transcriptase (Promega) using random primers. An Applied Biosystems 7500 equipment and HOT FIREPol EvaGreen qPCR Mix Plus (ROX) (Solis Biodyne) were used for RT-qPCR experiments. The specific primers were detailed in Supplementary Table 2. The temperature cycle program used was: 50 °C for 2 min, 95 °C for 15 min followed by 40 cycles of 95 °C for 15 s, 50–55 °C (depending of annealing temperature of primers used) for 30 s, and 72 °C for 45 s; followed by the dissociation step (95 °C for 15 s, 60 °C for 1 min, 95 °C for 30 s, and 60 °C for 15 s). Data analysis was performed with the 7500 system SDS v1.4.1 software (Applied Biosystems). Relative quantification of transcripts was determined by using the 2^−ΔΔCt^ method, with *gyrB* as the housekeeping control^61^.

### Quantification of promoter activity

To quantify promoter activity in biofilms, 100 μl/well of *S. aureus* cultures (OD_600_ = 0.05) were loaded in microplates and statically incubated at 37 °C for 24 h. After measuring the OD_595_ corresponding to growth, the GFP fluorescence (F) was detected (λ_exc_ = 485 nm, λ_em_ = 516 nm) with a fluorometer (FLx800 BioTek). To quantify promoter activity in planktonic cultures, *S. aureus* cultures set at an OD_600_ of 0.05 were grown at 37 °C and 200 rpm to measure GFP fluorescence every 2 h after measuring DO_595_. Promoter activity was indicated as the relationship (F/OD) to minimize variations owing to changes in OD between experiments.

### Analysis of proteins by MALDI-TOF–MS

Colonies of the Newman strain cultured on TSA and TSA plus SAL were resuspended in a mixture of 300 µl of distilled water and 900 µl of pure ethanol. Supernatants were discarded and pellets were resuspended in a mixture of the same volume (20 µl) of formic acid and acetonitrile. The supernatant of the previous step contained the cytosolic proteins. MS analysis were carried out with alfa-cyano-4-hydroxycinnamic acid matrix in 50% acetonitrile and 2.5% trifluoroacetic acid. One µl of the protein extracts was loaded onto the target and overlaid with 0.9 µl of matrix solution before drying. Continuous mass spectra were obtained with a Microflex LT mass spectrometer (Microflex, Bruker Daltonics, Massachusetts, USA) within a mass range of 2000–20,000 Da. Identification MS were first analyzed with MALDI Biotyper 2.0 v3.1 software (Bruker Daltonics, Massachusetts, USA), and reference library 3.1.1.0 (Bruker Daltonik GmbH, Bremen, Germany). Only spectra identified at the species level were considered for further analysis. The dataset contains 19 mass spectra from Newman strain growing in both conditions, including both technical (3) and biological replicates (3) which have been deposited in the https://github.com/MarManLed/Newman platform. Mass spectra were read as fid/aqus files with MALDIquantForeign (v0.10)^62^, and they were processed using MALDIquant (v1.16.2)^63^ R package. MALDI-TOF–MS data were analyzed either as intensity/peak or binary categorized/peak. The differentially significant proteins among the groups were analyzed. Intensity signals categorization was performed with the Binda R Studio package^64^. Programmed feature (peaks) selection was performed in the dataset seeking for biomarkers of each growing condition by the Binary Discriminant Analysis (BDA) algorithm. The algorithm output the *t-*score (Class means vs. Pooled mean) of each peak. The sign of the *t*-score pointed out the presence (positive *t*-score) or absence (negative *t-*score) of that peak. A significance level of 95% was achieved if the *t*-score was equal or higher than 2.015 or equal or less than − 2.015 (degree of freedom = 5).

### Proteolytic, hemolytic activity and colony spreading on agar plates

Ten μl of mature biofilm supernatants were loaded into spots on TSA plates supplemented with sheep or rabbit blood at 5%, which permits observation of hemolytic activity of δ- and α-hemolysins, respectively. Also, 10 µl of the same supernatants were loaded into TSA plates supplemented with 10% milk to show proteolytic activity. After 18 h incubation at 37 °C the diameters of hemolytic or proteolytic halos were measured. To determine the spreading ability of *S. aureus* colonies, 10 µl of overnight cultures of grown with or without SAL were loaded on LB agar plates supplemented with or without 2 mM SAL. The colony spreading ability was observed after 24 h of incubation at 37ºC and the area of spreading was quantified with the Image J v1.52p software (available at https://imagej.nih.gov/ij^65^.

### Molecular docking study

To study the molecular docking between the LytR domain of AgrA (PDB entry: 3BS1) and SAL, the AutoDock4 v4.2.6 (http://autodock.scripps.edu) and the AutoDock Vina v1.1.2 (http://vina.scripps.edu) software were used^28,29^. Center grid position was fixed by calculating the mass center of AgrA and the form of the grid was defined as a rectangular hexahedron (52.5 Å × 67.5 Å) to cover the whole protein domain and allow SAL to adopt any conformation. Energy maps were obtained for each kind of SAL atom with a 0.375 Å resolution. One hundred independent molecular docking experiments were carried out with each software and the results were grouped using a Root Mean Square Deviation (RMSD) of 2 Å. The Lamarckian genetic algorithm (LGA) was used with AutoDock4 and the initial population was fixed in 350 with a number of energy evaluations of 10 × 10^6^ and a maximum number of generations of 2.7 × 10^4^. For AutoDock Vina default conditions were used. The parameters for local searching of the LGA kept the default values for both software packages utilized.

### Statistical analysis

Non-parametric data were analyzed with the Mann–Whitney test while normal distribution data were compared with the unpaired *t*-test by using the Graphpad software (v6.0; GraphPad Prism). For spreading area determination, statistical analyses were performed with one-way ANOVA and the Holm’s test for multiple comparisons. Values of *p* < 0.05 were considered significant.

## Supplementary Information

Supplementary Information

## Data Availability

The datasets generated during and/or analyzed during the current study are available from the corresponding author on reasonable request.
