# Supplementary material for: Salicylic acid stabilizes Staphylococcus aureus biofilm by impairing the agr quorum-sensing system
Source: Sci Rep. 2021 Feb 3;11:2953. doi: 10.1038/s41598-021-82308-y (PMC7858585; doi:10.1038/s41598-021-82308-y)
Supplement: Supplementary file 1 — Supplementary Information [file 41598_2021_82308_MOESM1_ESM.docx]

**SUPPLEMENTARY MATERIAL**

**Salicylic acid stabilizes *Staphylococcus aureus* biofilm by impairing the *agr* quorum-sensing system.**

Cristian Dotto, Andrea Lombarte Serrat, Martín Ledesma, Carlos Vay, Monika Ehling-Schulz, Daniel Sordelli, Tom Grunert, Fernanda Buzzola.

**Supplementary Figures, Tables and Notes**

**Supplementary Figure 1. Growth curve of the Newman and Newman *agr* strains cultured in the presence of SAL.** Bacteria were cultured in TSBg with or without 2 mM of SAL and tetracycline (5 µg/ml) addition to Newman *agr* mutant. Growth was monitored at different times through OD_600_ determination. Each dot represents the mean ± SEM of 3 independent experiments.

**Supplementary Figure 2. SAL affects *S. aureus* biofilm dispersion.** Each bar represents the mean ± SEM of 4 independent experiments. After different times (3, 6, 24 or 48 h) of static incubation in presence or absence of 2 mM SAL, the planktonic biomass of the supernatant was measured at the OD_595_ nm and the biofilm biomass was assessed by crystal violet staining. Statistical analysis was performed with the Mann-Whitney test.

**Newman**

**Newman**

**+ 2mM SAL**

**Newman *agr***

**α-hemolysin**

**δ-hemolysin**

**Proteolytic activity**

**Hemolytic activity**

**Newman *agr* + 2 mM SAL**


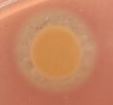

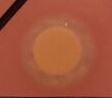

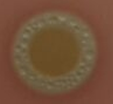

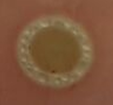

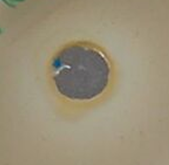

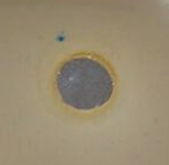

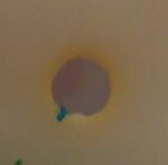

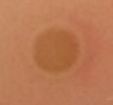

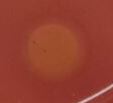

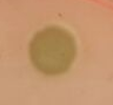

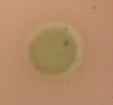

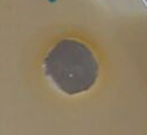


**Supplementary Figure 3: Hemolytic and proteolytic activity of supernatants of *S. aureus* biofilms.** Pictures representatives of TSA plates supplemented with rabbit or sheep blood at 5% (α-hemolysin and δ-hemolysin activity, respectively), or with 10% milk (proteolylic activity). Proteolytic activity is observed as a clearance-zone.


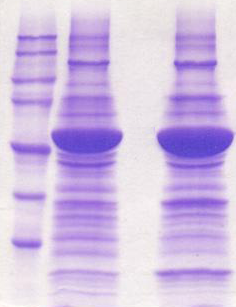


**Emp**

**MW**

**225**

**150**

**100**

**75**

**50**

**35**

**25**

**(KDa)**

**SAL [mM]**

**2**

**0**

**Eap**

**Ssl7**

**II**

**I**

**III**

**IV**

**V**

**VI**

**Supplementary Figure 4. Effect of SAL on surface proteins.** SDS-PGE of surface proteins extracted from 24 h biofilms of Newman strain formed with 2 mM of SAL. Each lane was loaded with the same amount of protein extracts. Long arrows indicate Eap (bands I and II), Emp (bands III and IV) and Ssl7 (bands V and VI) proteins identified by MALDI-TOF-MS/MS. Short arrows indicate other bands with change in presence of SAL. The left lane corresponds to the molecular weight marker (MW). Surface proteins were extracted, separated by SDS-PAGE and identified by MALDI-TOF-MS/MS following the procedures described in Dotto et al (2017) [1].

**Supplementary Table 1. MALDI-TOF-MS/MS**

| **Band No.** | **Protein name**  **(Uni-Prot accession No.)** | **MASCOT search results** | **MW (KDa) theor.** | **Probability based MASCOT score** | **Matched peptides MS/MS** | **Range** | **Peptide sequence** |
| --- | --- | --- | --- | --- | --- | --- | --- |
| I | Eap  (Q5HEI2) | gi\|57650691 Mass: 77013 Expect: 7e-22 Alternative name: Map protein [*Staphylococcus aureus* strain COL] | 65 | 283 | 7 | 669-676  375-394  477-497  49-72  49-79  268-299  267-299 | R.VFKATDIK.K  R.YVPYTIAVNGTSTPILSDLK.F  K.ADSYVPYTIAVNGTSTPILSK.L  K.VHVPYAITVNGTSQNILSSLTFNK.N  K.VHVPYAITVNGTSQNILSSLTFNKNQNISYK.D  R.NYQVPYSINLNGTSTNILSNLSFSNKPWTNYK.N  K.RNYQVPYSINLNGTSTNILSNLSFSNKPWTNYK.N |
| II | Eap  (Q5HEI2) | gi\|417649105 Mass: 77012 Expect: 7e-22 Alternative name: Map protein [*Staphylococcus aureus* strain 21189] | 65 | 283 | 7 | 669-676  375-394  477-497  49-72  49-79  268-299  267-299 | R.VFKATDIK.K  R.YVPYTIAVNGTSTPILSDLK.F  K.ADSYVPYTIAVNGTSTPILSK.L  K.VHVPYAITVNGTSQNILSSLTFNK.N  K.VHVPYAITVNGTSQNILSSLTFNKNQNISYK.D  R.NYQVPYSINLNGTSTNILSNLSFSNKPWTNYK.N  K.RNYQVPYSINLNGTSTNILSNLSFSNKPWTNYK.N |
| III | Emp  (Q5HHM6) | gi\|57650133 Mass: 38518 Expect: 1.8e-14 Alternative name: secretory extracellular matrix and plasma binding protein [*Staphylococcus aureus* strain COL] | 35 | 209 | 7 | 218-227  46-54  242-252  184-194  206-217  110-129  312-332 | K.FVTPGHASIK.I  K.IIPAYDEFK.N  K.VIPPYGHNSHR.M  K.HFIVKPSESPR.Y  K.HHFAVPGYHAHK.F  K.EINNRVNVATNNPASQQVDK.H  K.NVNYQYAVPSYSPTHYVPEFK.G |
| IV | Emp  (Q5HHM6) | gi\|57650133 Mass: 38518 Expect: 8.7e-05 Alternative name: secretory extracellular matrix and plasma binding protein [*Staphylococcus aureus* strain COL] | 35 | 225 | 3 | 45-56  241-254  183-196 | K.IIPAYDEFK.N  K.VIPPYGHNSHR.M  K.HFIVKPSESPR.Y |
| V | Ssl7  (A0A0H3KAV3) | gi\|57651309 Mass: 25631 Expect: 1.4e-14 Alternative name: superantigen-like protein [*Staphylococcus aureus* strain COL] | 18 | 180 | 9 | 148-154  44-53  92-104  67-79  141-154  90-104  122-139  61-79  183-203 | K.DFYYISK.E  K.HYYNKPILER.K  K.DGENSNIDVFILR.E  K.HYLEVTVGQQHSR.I  K.DNEDVLKDFYYISK.E  K.FKDGENSNIDVFILR.E  K.SNSVQYIDYINTPILEIK.K  K.YTDEGKHYLEVTVGQQHSR.I  K.QGQITITMNDGTTHTIDLSQK.L |
| VI | Ssl7  (A0A0H3KAV3) | gi\|57651309 Mass: 25631 Expect: 4.4e-15 Alternative name: superantigen-like protein [*Staphylococcus aureus* strain COL] | 18 | 215 | 12 | 44-53  92-104  67-79  141-154  90-104  122-139  122-140  61-79  9-31  36-53  183-203  183-206 | K.HYYNKPILER.K  K.DGENSNIDVFILR.E  K.HYLEVTVGQQHSR.I  K.DNEDVLKDFYYISK.E  K.FKDGENSNIDVFILR.E  K.SNSVQYIDYINTPILEIK.K  K.SNSVQYIDYINTPILEIKK.D  K.YTDEGKHYLEVTVGQQHSR.I  K.ASLALGMLATGVITSNVQSVQAK.A  K.QQSESELKHYYNKPILER.K  K.QGQITITMNDGTTHTIDLSQK.L  K.QGQITITMNDGTTHTIDLSQKLEK.E |

**Supplementary Figure 5. Molecular dynamic simulation of AgrA-SAL complex.** They show RMSD values (root mean square deviation) over time. **(a)** It shows the fluctuations corresponding to the protein backbone, reaching the stabilization from the 50 ns. **(b)** It shows the fluctuations owing SAL present in site number 4 of AgrA, reaching the stabilization from the 65 ns.

**Supplementary Figure 6. Interaction between SAL and site 4 of AgrA.** They show the regulator AgrA harboring SAL in site number 4 (denoted with ellipses) overlapping a DNA double helix, **(a)** front and **(b)** inverted, respectively. The Tyr residue is denoted with a green color “van der Waals” representation and the double helix with a “Surface Accessibility Solvent Area” representation, with N, O, C and P in blue, red, cyan and gold, respectively. The images were made with Visual Molecular Dynamics (VMD) v1.9.2 ([http://www.ks.uiuc.edu/](https://www.ks.uiuc.edu/)). VMD is developed with NIH support by the Theoretical and Computational Biophysics group at the Beckman Institute, University of Illinois at Urbana-Champaign.software support [2].

**Supplementary Note 1. Simulation of molecular dynamics study.** Molecular dynamics simulations were carried out by the Bioinformatics Service (IQUIBICEN, Buenos Aires, Argentina). AgrA structure and the one of the complexes obtained by docking with SAL in a saturated concentration of 260 mM, were solvated with a water molecules box TIP3P with a truncated octahedral form. Those structures were parameterized with the force fields ff14SB and AM1 by using LEaP module of AMBER package [3]. All stages of the simulation were performed with PMEMD.CUDA module of AMBER which corresponds to a new implementation in GPU (Graphics Processor Unit) of PMEMD (Particle Mesh Ewald Molecular Dynamics) module [4]. The general protocol comprised an optimization step where 10 and 4990 cycles of Steepest Descent and Conjugate Gradient algorithms were used, respectively. The thermalization step comprised the first stage of heating from 0 to 10 K for 10 ps with a force constant of 50 kcal/mol applied to all protein atoms and a second stage of heating from 10 to 300 K for 90 ps with a force constant of 10 kcal/mol. For the thermalization step, a constant volume of the system was considered and the Berendsen thermostat was used. Next, the balancing step at particles number, temperature and, pressure constant was carried out by a simulation of 5 ns at 300 K by using Langevin thermostat and applying a 1 atm pressure to the system with the Berendsen barostat. A force of 1 kcal/mol was applied to limit the protein atom’s mobility during the process. Finally, for the production step at particles number, volume and temperature constant, the Berendsen thermostat was used by keeping the temperature at 300 K and shutting down the barostat to generate similar conditions to particles number, volume and temperature constant and eliminating any restriction for protein atoms. The simulation time was 100 ns and the coordinates for each atom were registered every 2 fs. In all stages, periodic conditions of contour, Ewald sum method and a cutting value of 10 Å for non-union interaction, were used. In all cases where Berendsen thermostat was used, a coupling constant of 5 was used while a constant of 1 was used for Langevin thermostat.

**a**

**b**

**Supplementary Figure 7. Endogenous ROS production by *S. aureus* exposed to SAL.** Intracellular ROS quantified by OD_540 nm_ relative of growth measured to OD_G_ from biofilms **(a)** or planktonic cultures **(b)** of Newman strain grown with different concentrations of SAL during 24 h. Each bar represents the mean ± SEM from 3 independent experiments.

**Supplementary Note 2. Quantification of reactive oxygen species (ROS) in biofilm.** ROS were quantified in *S. aureus* biofilms as described in Becerra et al (2002) [5]. Thus, after measuring OD_G_ of 24 h biofilms formed with or without SAL, culture media were removed and replaced by a solution formed with 100 µl of NBT (1 mg/ml) and 100 µl of TSBg. After incubation for 15 min at 37ºC, 50 µl of DMSO was added and the OD at 540 nm (OD_540 nm_) was determined. OD_540 nm_ values were normalized to its respective OD_G_ values.

**Supplementary Table 2. Strains and plasmids of *S. aureus*.**

| **Strains** | **Relevant characteristics** |
| --- | --- |
| BRA | MRSA strain representative of brazilian clone, *agr* type I [6] |
| CBS | MRSA strain representative of cordobés clone, *agr* type II [6] |
| MW2 | MRSA strain, *agr* type III [7] |
| RN8540 | MRSA strain, *agr* type IV [6] |
| Newman | Strain ATCC 25904; *agr* type I [8] |
| Newman *agr* | Newman *agr*::*tet* [9] |
| Newman_P3_*_agr_* | Newman with pALC1743 [10] |
| Newman_P_*_spa_* | Newman with pALC1741 |
| Newman_Pnull_ | Newman with pALC1484 [11] |
| **Plasmids** | **Relevant characteristics** |
| pALC1484 | Derivative from pSK236 with *gfp*_uvr_ without promoter preceded by a ribosome binding site of *S. aureus*, Cm^R^ [11] |
| pALC1741 | pALC1484 with *spa* promoter merged to *gfp*_uvr_  [12] |
| pALC1743 | pALC1484 with P3 *agr* promoter merged to *gfp*_uvr_ [13] |

**Supplementary Table 3.** **Primers used for qRT-PCR.**

| **Primers** | **Sequence 5´- 3´** | **Source** |
| --- | --- | --- |
| *gyrB* | F: GGTGCTGGGCAAATACAAGT  R: TGGGATACCACGTCCGTTAT | [14] |
| RNAIII | F: TTCACTGTGTCGATAATCCA  R: TGATTTCAATGGCACAAGAT | This work |
| *agrA* | F: CATTTGCGAAGACGATCC  R: GATTATCAGTTGCGAGGG | This work |
| *agrC* | F: CTGGCCTACGTGATTA  R: GCTACTTACTTCATCGGG | This work |
| *psmα_1-2_* | F: TGGGTATCATCGCTGGCATC  R: TACTTACCAGTGAATTTCTC | [15] |
| *psmα_3-4_* | F: ATGGAATTCGTAGCAAAATTATTC  R: GCGAAAATGTCGATAATTGCTT | [15] |
| *psmβ_2_* | F: CTGCACAACAACATGATAG  R: TTACCTAGTAAACCCACAC | This work |

**Supplementary References**

1. Dotto, C. M. et al. The active component of aspirin, salicylic acid, promotes *Staphylococcus aureus* biofilm formation in a PIA-dependent manner. *Front. Microbiol .***23**, 8:4 (2017).
2. Humphrey, W., Dalke, A., & Schulten, K. VMD: visual molecular dynamics. *J. Mol. Graphics* **14**, 33–38 (1996).
3. Case, D.A. et al. The Amber biomolecular simulation programs. *J. Computat. Chem.* **26**, 1668-1688 (2005).
4. Salomon-Ferrer, R., Goetz, A.W., Poole, D., Le Grand, S. & Walker, R.C. Routine microsecond molecular dynamics simulations with AMBER on GPUs. 2. Explicit solvent Particle Mesh Ewald. *J. Chem. Theory Comput.* **9**, 3878-3888 (2013).
5. Becerra, M.C. & Albesa, I. Oxidative stress induced by ciprofloxacin in *Staphylococcus aureus*. *Biochem. Biophys. Res. Commun*. **297(4)**, 1003-1007 (2002).
6. Lattar, S.M. et al. Molecular fingerprinting of *Staphylococcus aureus* isolated from patients with osteomyelitis in Argentina and clonal distribution of the *cap5(8)* genes and of other selected virulence genes. *Eur. J. Clin. Microbiol. Infect. Dis.* **31**, 2559–2566 (2012).
7. Baba, T. et al. Genome and virulence determinants of high virulence community-acquired MRSA. Lancet. **359**, 1819–1827 (2002).
8. Duthie, E & Lorenz, L. Staphylococcal coagulase : mode of action and antigenicity. *J. Gen. Microbiol.* **6**, 95–107 (1952).
9. Wolz, C., McDevitt, D., Foster, T.J. & Cheung, A.L. Influence of *agr* on fibrinogen binding in *Staphylococcus aureus* Newman. *Infect. Immun*. **64**, 3142–3147 (1996).
10. Barbagelata, M. et al. Auxotrophic mutant of *Staphylococcus aureus* interferes with nasal colonization by the wild type. *Microbes Infect.* **13**, 1081–1090 (2011).
11. Manna, A. & Cheung, A. L. Characterization of *sarR*, a modulator of *sar* expression in *Staphylococcus aureus*. *Infect. Immun.* **69**, 885–896 (2001).
12. Kupferwasser, L. et al. Salicylic acid attenuates virulence in endovascular infections by targeting global regulatory pathways in *Staphylococcus aureus*. *J. Clin. Invstigation*. **112**, 222–233 (2003).
13. Kahl, B. et al. *Staphylococcus aureus* RN6390 replicates and induces apoptosis in a pulmonary epithelial cell line. *Infect. Immun.* **68**, 5385–5392 (2000).
14. Alvarez, L. et al. Salicylic acid diminishes *Staphylococcus aureus* capsular polysaccharide type 5 expression. *Infect. Immun*. **78**, 1339–1344 (2010).
15. Kaito, C. et al. Transcription and translation products of the cytolysin gene *psm-mec* on the mobile genetic element *SCCmec* regulate *Staphylococcus aureus* virulence. *PLoS Pathog*. **7(2)**, e1001267 (2011).
